# Monthly Periscope

**Published:** 1853-12

**Authors:** 


*Monthly Periscope.— Opium Eating.*—The New York Medical Times has an article, written by an opium eater, describing the effects of that drug when taken to excess, both bodily and mental. We have needed something of this kind. The effect of De Quincey's wonderful revelations was to charm the reader, and many an infatuated being has become an opium eater with the design of becoming also a De Quincey. The author of Suspiria dwelt also too much upon his mental sensations, and giving to the description all the native beauty and energy of his wonderful powers, he has thrown about the subject a charm, which conceals the real horrors which beset the opium-eater. And even his account of the pains of opium is so grand in conception, so magnificent in detail, that many romantic youths have eaten the drug to be themselves the heroes of such dreams.

Such is not the case with the present writer. His description of the sufferings he endured in several futile attempts to abandon the poison, is pathetic from its very plainness and minuteness of detail. One curious fact he mentions, and which we believe is true of most opium eaters, is that the increase of the quantity consumed was rapid in his case up to half an ounce of the gum weekly, but that once arriving at that point, it was sufficient to produce all the pleasurable effects to be desired from its use, and there was no further increase for years. He also states that on reaching this point the pleasurable feelings consisted merely in a negation of discomforts, by which he was enabled to perform his ordinary routine of duty with perhaps increased mental vigor.

He makes a nice but truthful distinction between this mental vigor and the disposition to employ it. Place the opium-eater in a situation where exertion is necessary, or unavoidable, and he will acquit himself with more energy and force than when in health; but he will never seek an opportunity for distinction or effort, and never place himself in the way of labor. In a word, his will is wanting.

This well accords with De Quincey's closing paragraph of the Pleasures of Opium, where (we have not the book at hand and cannot quote) sitting at his window from sunset to sunrise, he beheld the ocean brooded over by everlasting calm, typifying the peace which possessed him, a peace which brought forgetfulness of wrongs, and present rest instead of hope: which reconciled his love for the living, with the dishonors of the grave, and knew no source of ambition, of hope, of regret, or of sorrow. But we are growing metaphysical.

*Typhoid Fever and Typhoidism.*—This is the taking title of a translation from the French of M. J. B. Cayol, which we find in the Virginia Medical and Surgical Journal, for October. It has a startling motto prefixed.

" *Les systémes en médecines sont des idoles auxquelles on sacrifie des victimes humaines.*"

Typhoid*ism*, we may say for the benefit of the uninitiated, is that proclivity now manifested in France, and for aught we know in this country, to consider all fevers as typhoid. It is, then, an affection of the practitioner, rather than the patient. The physician laboring under this condition, is said to be *typhoidian.* Having thus established our nomenclature we shall proceed to state M. Cayol's argument. The typhoidian is necessarily a fatalist. Any fever lasting more than seven or eight days is christened typhoid, and the doctor bows his head in submission, awaits the decrees of destiny, and says "Allah is great." It is a peculiar typhoidian notion that typhoid fever is imperturbable—that do what you please it follows its course. Treatment is indifferent. Whether by blood-letting, by purgatives, by quinine, by opium, or nothing at all, it makes no difference. Such a percentage must die.

We have not space to lead our readers through all this spicy article's sharp points, neither have we any disposition to copy it entire. One would suppose from the motto at the head, that M. Cayol had a most decided aversion to theory. We are not sure but he has, but he also speaks in most contemptuous terms of statistical research. Statistics, he assures us, are mere empiricism, a reference to blind experience as the guide of our treatment. Then, immediately resorting to the method he condemns, he finds a wonderful increase in the mortality of the age, and for this increase typhoidism is responsible. He next charges upon the typhoidians an attempt to account for an increased prevalence of typhoid, by a corresponding decrease of small-pox.* He denies the existence of any increased frequency of typhoid, and asserts that the idea originates in an error of fashionable diagnosis, which calls all enteric inflammation, or continued fever, typhoid.

We have noticed thus briefly this attack upon the doctrines of Louis, as an indication that we are to have a change of opinion, or at least a renewed discussion on this subject. But it seems rather rash to deny that there is a real increase of typhoid fever in this country, whatever may be the case in France.

There is something in the piquant tone, and spicy paragraphic style of M. Cayol's article, which gives the idea of a personal feeling against M. Louis. If this is the case we shall probably hear but little more of typhoidism and typhoidians.

*Volitional control of involuntary muscles.*—One of the most curious experiments in clairvoyance, or animal magnetism, is that by which the pulse of the subject is accelerated to an unusual frequency. This has long been one of the triumphant experiments of peripatetic lecturers, and one which has hitherto been unexplained.

A non-medical friend, who is remarkable for his shrewdness in detecting the impositions of these latter-day "sciences," and who is known to our readers by the cognomen of "Shadrach Barnes," in connection with some ludicrous exposures of spirit-rappings, has ascertained the method by which this phenomenon is produced; and by the aid of an accomplice in one of his whimsical, but well-meant deceptions, succeeded in gulling a large audience of all the learned pundits, both medical and laical, of a certain pleasant village, into the belief that this increased frequency of the pulse was a mesmeric condition. Of course our friend maintained the deception only long enough to accomplish his purpose of throwing ridicule upon the science.

The deception was managed this wise: The "subject," maintaining an unchanged countenance, induces contractions of the abdominal muscles and

* We have, in a previous number, alluded to this curious doctrine. Assuming a certain law of compensation in disease, they make variola a substitute for typhoid, and *vice versa*.

sphincters—in a word, he strains as if going to stool. A continuation of this straining effort will, in a few minutes, increase the frequency of the pulse some twenty or thirty beats in a minute.

This method is not irrational, or unscientific. The recent researches of Hubert Luschka, of Tubingen, on the anatomy of the phrenic nerve, explain in some measure its mode of operation. Without going into a detail too tedious for our purpose, we will state that the phrenic nerve is distributed to portions of the heart, and to the peritoneum of the anterior abdominal parietes, as well as to the diaphragm. The connections of the phrenic with the sympathetic nerve, are very intimate. It should also be recollected that the phrenic is a spinal nerve, and our premises are complete.

Voluntary motion always increases the motion of the heart, and it is well known that the abdominal organs are also much influenced as to activity by voluntary motion, running, or other bodily exercise. Now a volition confined to abdominal organs, or muscles, alone, is impossible, as the phrenic is one of the nerves of that region, and a reflex action is conveyed to the heart by the connections of the phrenic with the par vagum. Aside from this supposed reflex action, there is a direct action. The phrenic is a cervical nerve, communicating with those other cervical nerves which control the abdominal muscles, and it supplies the diaphragm. Any volition directed to the diaphragm must therefore reach the heart and affect its motion correspondingly.

Another equally curious power is that of controlling the motions of the iris, also considered a mesmeric phenomenon. But every person has this power as clearly as the clairvoyant—and for the method of its exercise we are also indebted to our friend before-mentioned.

Let a person fix his eye steadily on any small object which is of a light color, or better still, a luminous body. After gazing steadily at it *without relieving the eye by winking*, the iris will slowly contract, and remain contracted. Then turn the eye, or better, direct it beyond to some large object of a darker or cooler shade, and the iris will dilate correspondingly. The purposes of deception can be better attained by gazing at a small body not marked by light, and fixing the gaze until the retina is confused, when the iris will contract to relieve it, thus acting vicariously to the eyelids. Then as before, by choosing a larger field of vision you relieve the retina, and the pupil dilates.

Lastly, we will mention the loss of pulsation at the wrist which accompanies the rigid condition of the arm produced by mesmerists. This is owing to pressure of the muscles upon the arteries above, and can be produced by any person of sufficient muscular power.

*Thoughts on the Rationale of Symptoms referable to Nervous Sympathy.*—When an individual has toothache from some irritation of the nervous pulp of a single tooth, it is not uncommon to find that the pain is diffused over a large area. Acute and lancinating pains are felt in the temple, and over the whole affected side of the face, the eye becomes suffused, and the cheek swollen, or tender to the touch.

In this familiar instance we have the type of a large class of symptoms usually called sympathetic pains. It is simply an expression of the fact, that an impression of unusual force being applied to a given point on the periphery of a nerve, will convey a sensation to other parts supplied by the same nerve. In the example quoted, we have the whole periphery of the fifth nerve answering to an impression upon a single point. The eye—that is, the conjunctival membrane, becomes suffused; the iris will also contract as if under the stimulus of a strong light.

Now as the conjunctiva and iris are supplied by the ophthalmic branch of the fifth pair, and the teeth of the lower jaw by the infra-maxillary, it becomes evident that this impression must have been directed to the cranial cavity, or at least as far as the casserian ganglion, and thence reflected through the ophthalmic branch to the organ of sight.

That there is a still more intimate connection of sensory nerves, is evident from sundry other circumstances.

The first of these to which we shall allude, is, like the second, confirmatory of the existence of a communication between the retina (second cranial nerve) and the ophthalmic branch of the fifth pair. This connection is formed through the filament of the nasal branch of the ophthalmic, communicating with the lenticular ganglion, and thence through the ciliary nerves, to the choroid coat overlying the retina.

In the diagnosis of cataract, it is usual to cover the eye with the hand. If by this means *total* darkness is substituted for the glimmer of light usual in cataract, it is considered as evidence that the retina is sensitive. But this is liable to error, as the sensation of light may be conveyed to the brain entirely through the supra-orbitar branch of the ophthalmic. The hand then, in covering the eye should be applied also to the forehead over the distribution of that nerve.

The second phenomenon in this connection, and having a practical bearing, is, that irritation of the dental nerves may produce the greatest intolerance of light, without any manifestation of the cause by pain in the tooth affected.

Some years ago, we met, in some one of the medical periodicals, with a report of two cases, which, after prolonged search, we are now unable to find. Of one of these cases our recollection is sufficiently distinct. A cashier of a bank in Georgia, after prolonged application to accounts, suffered from extreme intolerance of light, and finding no relief, came to Philadelphia for treatment. So acute was the sense of vision, that though his room was darkened to the utmost, the presence of any light colored substance was intolerable; and on one occasion the entrance of his negro nurse, having on a white apron, excited him almost to fury,—his temper, though usually mild, being extremely irritable during this attack. No appearances of disease were presented by the eye. By what means attention was directed to the teeth we do not recollect; but on the removal of a bicuspid of the upper jaw, the intolerance of light ceased. Another case, analogous in symptom, is embodied in the same report.

Probably few cases on record illustrate so strikingly the *modus operandi* of sympathy. It is believed that all cases of pains occurring at a distance from the part affected, are referable to nervous communication, direct or indirect, and that when no such communication can be traced, it is owing to deficient anatomical knowledge. That we have very much yet to learn of the anatomy of the nervous system, is evident from many facts. The phrenic nerve has never yet been traced near enough to its terminal branches, to ascertain whether or no it ends in loops.

Other sympathetic pains in the fifth pair are less easily explained. It not unfrequently happens that facial neuralgia depends upon gastric, hepatic, or enteric irritation. Why an affection so distant should single out this nerve as its point of manifestation is scarcely explicable. A careful consideration of all the phenomena connected with this point may afford some light. Although the portio-dura of the seventh pair is the nerve which controls the movements of the superficial facial muscles, a closer research into the ordinary expressions of pain will show that the fifth has much to do with this phenomenon. The corrugator supercilii is the muscle which conveys the earliest expression of pain, by knitting together the eye-brows; the clenching of the teeth, and the drawing down of the lower lip, as well as the contraction of the pupil, which accompanies severe pain, are indications that to the fifth nerve, which supplies these parts, and the muscles which move them, is committed the function of the expression of pain, as well as to the portio-dura. Now, as all pains, wherever located, thus express themselves in the countenance, it is not difficult to conceive, that by an error of sensation, pain itself may, like its expression, be conveyed to this nerve.

This phenomenon, though in itself inexplicable, is not without its analogies in perversion of nervous function. Thus, chloroform, which is usually directed to the sensory nerves, sometimes changes its habitat, and affects only the motor nerves, so that a patient, under chloroform, remains motionless while suffering the acutest pain from a surgical operation. A precisely similar instance is found in the action of some blood poisons. Urea usually involves first, in its effects, the sensory nerves, producing painless coma and stupor. Yet it sometimes happens that its action is expended upon motor nerves, producing paralysis, while the sensory nerves are in full action. No nerve in the body is entirely isolated from its neighbors; and it is easy to understand that abdominal irritation may be conveyed to the fifth pair by the medium of either the sympathetic or the phrenic.

Another fact relative to the fifth pair is worthy of consideration. Of all sensory nerves this one is most constantly exposed to atmospheric contact and vicissitudes. No nerve receives so many impressions. Its gustatory branch is continually subjected to the most diverse of sensations. It is, consequently, very often a weak point in the chain of nervous connection, and thus manifests reflected pain more readily than its fellows.

The consideration of the deranged sensations of the fifth pair would occupy more space than our ability, or the scope of this paper, will permit. It is our object to account rationally, as far as possible, for all transmitted pains by anatomical and physiological facts. In so doing we shall avoid all hypothetical argument, and endeavor to derive some practical results from this intricate but useful branch of study.

In accordance with this plan we turn next to the occipital and vertical portion of the head. We are often called upon to prescribe for various degrees of deranged sensation in this part. Sometimes we have only a feeling of constriction, sometimes a "cobweb sensation," and oftener a positive neuralgic pain. This pain is in almost every instance sympathetic with the condition called spinal irritation; and the point of tenderness in the vertebral column is usually in the upper cervical region: a fact sufficiently accounted for by the distribution of the great occipital nerve, which is a branch of the third cervical, and is accompanied by filaments from the sub-occipital, and second cervical. The practical indication is here evident. The most active treatment over the painful part, is not uncommonly useless, while very moderate counter-irritation over the origin of the nerve effects a speedy cure.

*The phrenic nerve.* To this offset from the cervical plexus are due a large number of sympathetic pains. The first, and most common of these, is that acute pain in the posterior acromial region which accompanies hepatic disorder. Accordingly we find pain in the *right* shoulder enumerated as one of the symptoms of hepatic inflammation. But as every observer has noticed, this pain is not peculiar to the right shoulder, it being as often found in the left. This is owing to the decussation of the phrenic nerve, its terminal branches crossing the median line from side to side, so that an irritation of the left hypochondrium may cause pain in the right shoulder. The phrenic nerve in the neck gives an offset to a sensory branch of the fifth cervical, going to the integument of the shoulder, and hence the connection between these distant parts.

The decussation of the phrenic nerve deprives pain in the shoulder of any diagnostic value as a symptom of hepatic disease. All portions of the nerve do not decussate; and before reaching the diaphragm, it gives offsets to the right auricle, and ascending vena cava. Owing to this distribution, pain in the shoulder may be due to irritation reflected from either the liver, the diaphragm, the peritoneum of the left hypochondrium, or (a portion of the distribution we have omitted to mention) the costal pleura. Thus while this pain is not diagnostic of any particular lesion, it points to a number of organs, either of which may be the point involved. One further practical conclusion may be drawn. Pain in the shoulder is a common symptom of pleuritis, even if the inflammation be low down in the thorax. But it is *not* a symptom of pneumonia unless the costal pleura be involved, inasmuch as the pulmonic pleura is supplied by the vagus, and not at all by the phrenic; while the parietal pleura is largely supplied with offsets from the phrenic.

Dr. Hubert Luschka, of Tubingen, Germany, who has recently published a learned and valuable monograph on the phrenic nerve, which adds largely to our anatomical knowledge, has pointed out some practical points as to remedial measures. The vagus nerve sends a much smaller nervous supply to the pulmonic pleura than does the phrenic to the parietal; and to this fact of free supply from a sensory nerve in direct communication with the spinal cord and brain, he attributes the acute pain which marks parietal pleuritis, and the aggravation of the suffering by movement of the walls of the chest. Many of the shoulder pains accompanying pleuritis, ordinarily called rheumatic, he considers as depending on disease of the pleura involving the terminal branches of the phrenic. We quote from him a paragraph relative to remedial measures:

"The place of origin of the nerve points out the spot at which derivative or remedial means may be applied for the relief of acute pain. There is no doubt that blisters, and narcotic remedies applied to vesicated surfaces, will act most efficaciously immediately above the clavicle, at the spot where direct branches of that nerve are found, whose ramifications are affected at the peripheral termination."

We cannot leave the subject of the phrenic nerve, without allusion to some symptoms occasionally present in phthisis pulmonalis, which implicate the action of the diaphragm. It sometimes happens that in this, and in cardiac disease, there is permanently embarrassed respiration, evidently dependent on deficient nervous supply to the diaphragm. On this point Mr. Luschka remarks as follows:

"I have repeatedly made the observation, that by the softening of tubercle and the destruction of the lung-substance united with the pericardial pleura, the phrenic was almost destroyed and interrupted in its continuity at a certain point. One instance of this I have preserved, in which the right phrenic was so surrounded in one part by calcareous tubercular material of the hardness of stone, that it was impossible to detach it."

The further pursuit of this inquiry requires more leisure than we now possess. We hope to find time before our next number, to consider some of the mysteries of uterine sympathy.

*Anatomical Dissections again.*—It is said that the defeat of Richard S. Williams, a candidate for State Senator in the city of New York, was owing to his declared opposition to the law legalizing anatomical pursuits. If this be true, we say, all honor to the medical men of that senatorial district!

We have no disposition to let *this* "subject" rest. It should be disinterred as often as our legislature bury it beneath the table. We are sure that none of its friends will object to such a violation of its grave. Write upon its tombstone, "Resurgam!"

The time for the opening of the legislative session is now drawing near, and we again take occasion to remind the county societies of their duty in urging the passage of the law we require. Hitherto the success of any efforts directed to this end, has depended upon the exertions of a few men who had sufficient *esprit du corps* to take the matter in hand. These early friends of the law now ask the co-operation of the profession at large, through their legal representatives, the county and state societies.

We have recently heard through the papers of an excitement near Syracuse, owing to a violation of the present enactment, and the arrest of some of those suspected of connection with it. Such occurrences will happen, and bring in their train disastrous consequences, so long as a love of science exists in the profession, unless our law-makers remove the good and sufficient excuse which now exists for them. That they can do only in the manner that we ask.

*Hygrometric Observations.*—We need make no apology to our readers for the space which the article of Mr. Blodgett occupies in our eclectic department. A careful perusal of it will go far to convince the inquirer that one great cause of epidemic influence has been neglected.

A few facts bearing on this question—and which of course were not within the range of a statistical paper—occur to us at this writing. Among the more valuable of the deductions made by the Registrar General of England, in his statistical record of the cholera in London, was the fact that the epidemic raged most fatally near the banks of the Thames, and that with the increase of distance from its banks, and of height above its level, there was a corresponding decrease of choleraic disease.

The fact that in these lower parts of cities is collected the poorer and more filthy part of the population, that the streets are there more dirty and the tenements badly ventilated, has drawn away attention from the atmospheric condition, to that of decomposition of organic matter.

It was the notion of the earlier investigators of the causes of malaria, that its three requisites were heat, moisture, and vegetable decomposition. The condition of moisture can only be ascertained by accurate hygrometric observation. The want of this has given rise to those opposite opinions which have been so ardently maintained. Thus, great heat alone is rarely, or perhaps never the cause of zymotic disease. During the past summer we had evidence of that, in the coincidence of unusual general health, with the most exhausting temperature. But in New Orleans the season was wet, heavy showers falling every morning, to be dried up by a burning noonday sun. Here we have the two conditions which Mr. Blodget deems necessary for an epidemic cause. At the north we had but one of them.

It has long been evident that vegetable decomposition does not sufficiently account for malarial disease. Were it the only cause, we should see its effects in every place where vegetation exists. Some of the most malignant malarial visitations on record, occurred where there was almost no vegetation, as in the instance of the Walcheren expedition.

But it happened that on that sandy, and apparently dry island, water was to be found anywheres a few inches beneath the surface. But it is probable that in most instances the moisture with which the atmosphere is charged, holds in solution some decomposing organic matter.

We hope another season to be able to compare the health of the city of Buffalo with the degree of humidity of the atmosphere. It is almost useless to hope that we may also have an accurate record of the diseases and deaths. The curse of political expediency hangs over medical progress in this direction. S. B. H.

